# Elevated Colon Cancer Rates Linked to Prior Appendicitis: A Retrospective Cohort Study Based on Data from German General Practices

**DOI:** 10.3390/jcm13082342

**Published:** 2024-04-18

**Authors:** Susann Steffes, Karel Kostev, Jörn M. Schattenberg, Hauke S. Heinzow, Miriam Maschmeier

**Affiliations:** 1 Department of Internal Medicine I, University Medical Centre of the Johannes Gutenberg-University, 55131 Mainz, Germany; joern.schattenberg@uks.eu; 2 Epidemiology, IQVIA, 60549 Frankfurt am Main, Germany; karel.kostev@iqvia.com; 3 Department of Internal Medicine II, Saarland University Medical Center and Saarland University Faculty of Medicine University, 66421 Homburg, Germany; 4 Department of Internal Medicine B, University Hospital of Münster, 48149 Münster, Germany; h.heinzow@bbtgruppe.de; 5 Department of Internal Medicine I, Krankenhaus der Barmherzigen Brüder, 54292 Trier, Germany; m.maschmeier@bbtgruppe.de

**Keywords:** appendicitis, colon cancer, carcinoma, incidence, appendectomy

## Abstract

**Background/Objective:** The association between appendicitis and colon cancer is not yet fully understood. Previous studies have shown contradictory results. Currently, no population-based data from Germany are available with regard to the incidence of colon cancer following appendicitis. This study investigated the association between appendicitis and the incidence of colon cancer in Germany. **Methods:** In this retrospective cohort study, the incidence of colon cancer was compared for patients with appendicitis and patients without appendicitis, matched for age, sex, index year, average annual consultation frequency, and comorbidity. The aim of the study was to explore the relationship between appendicitis and the incidence of colon cancer. The evaluation was carried out using logistic regression analyses. **Results:** The study included 49,790 people with and without appendicitis, with a median age of 41 years. During a follow-up period of up to 15 years, 1.04% of cases with appendicitis and 0.60% of cases without appendicitis were newly diagnosed with colon cancer, with some 36.4% of colon cancer cases diagnosed within the first six months after appendicitis. Regression analyses revealed a significant association between appendicitis and colon cancer, particularly in men and in the age groups 41–50 (HR: 10.30; 95% CI: 1.03–43.82) and 18–30 years (HR: 8.17; 95% CI: 1.03–64.58). **Conclusions:** The present retrospective cohort study suggests an association between appendicitis and the incidence of colon cancer in Germany. Based on our results, we recommend offering a colonoscopy or at least a stool test within 12 months after appendicitis, especially for 18–50-year-olds and >60-year-olds in good general health.

## 1. Introduction

The main cause of appendicitis is the obstruction of the lumen [1]. Depending on the age of the patient, obstruction is caused by follicular hyperplasia due to infection, fecal stones, or neoplasia, resulting in secondary infection and bacterial colonization [2,3].

The adenoma–carcinoma sequence is an established concept of the evolution of colon cancer. It covers about 60% of the development processes of colon carcinomas. Both hereditary and non-hereditary causes are listed as the main mechanisms of development [4,5].

Inflammatory processes, a low-fiber diet, and the composition of the intestinal microbiome, as well as age and obesity, are considered risk factors for colorectal cancer. It can take decades to progress from status zero, i.e., no mucosal changes, to invasive carcinoma. During this process, various mutations favoring degeneration accumulate. Inflammation, diabetes and obesity also have a negative impact on this process. This is due to increased levels of the insulin-like growth factor, chronic inflammation and oxidative stress. Nicotine also increases the rate of proliferation [6,7,8,9]. It is unclear to what extent the presence of several risk factors accelerates this process and how they reinforce each other.

In addition to the adenoma–carcinoma sequence, there is an alternative pathway of carcinogenesis that has recently been described. Neoplasms of the intestine arise from a precursor lesion known as a sessile serrated adenoma. These lesions are mainly found in the proximal colon and differ from the classic adenoma–carcinoma sequence due to their potentially faster carcinogenesis [4].

In this context, the extent to which an unhealthy appendix, appendectomy, and associated changes in the intestinal microbiome play a role in the development of cancer has been discussed repeatedly. Vitetta describes the appendix as a “safe microbiome house” that reflects and determines the health of its host [10].

The minimum duration for the development of colon cancer is estimated to be approximately five years, which is reflected in the intervals for colon cancer screening [11].

Almost 100,000 cases of appendicitis were diagnosed in Germany in 2021 [12]. Therefore, appendicitis—like colon cancer, with over 70,000 diagnoses in the same year—is a common disease [13]. The possibility of a connection between the two diseases has been discussed for decades. In 2006, Lai and colleagues showed a 38.5-fold increase in the likelihood of developing colon cancer after appendicitis [14]. Although this study was published over fifteen years ago and was followed by a number of smaller, single-center studies with confirmatory results [15,16,17], these have not yet been taken into account in the current German S1 guideline for the treatment of acute appendicitis. The guideline neither refers to an increased risk nor recommends standardized follow-up care [18].

Looking at the current state of research, a meta-analysis from 2020 criticizes the lack of robust data. Among other things, studies with comparison groups from the general population, as we are investigating in our study, are missing [19]. It should also be emphasized that the cancer of the cecum and proximal colon associated with appendicitis has a poor prognosis. Ramprasad P. Rajebhosale and colleagues attribute this to delayed diagnosis [17]. Despite the described knowledge of the association between appendicitis and colorectal cancer, as also described by Viennet et al. [20], there is a lack of compliance with recommended screening colonoscopies [21].

The motivation for this research was to investigate the correlation between colon cancer and appendicitis using data from a large patient database in the outpatient sector and to create a broader basis for recommendations regarding follow-up after appendicitis to ensure the early detection and treatment of colon cancer.

## 2. Materials and Methods

### 2.1. Database

This retrospective cohort study is based on data from the Disease Analyzer database (IQVIA), which contains drug prescriptions, diagnoses, and basic medical and demographic data obtained directly and in anonymized form from the computer systems of GP (general practice) and specialist practices [22]. IQVIA is a leading global provider of advanced analytics, technology solutions and clinical research services to the life sciences and healthcare industries [23]. The database covers around 3% of all general practices in Germany. The diagnoses analyzed were selected on the basis of ICD-10 codes (International Classification of Diseases). The ICD-10 codes used are K35–K37 for appendicitis and C18.0–18.7 and C.18.9 for the neoplasia of the colon.

The sampling method for the Disease Analyzer database is based on statistics from all the doctors in Germany published yearly by the German Medical Association. IQVIA uses these statistics to determine the panel design based on the four strata of specialist group, federal state, community size class, and physician age.

It has already been shown that the selection of practices included in the Disease Analyzer database is representative of general and specialist practices in Germany, and the database has served as the basis for previous studies focusing on cancer [22,24,25].

### 2.2. Study Population

**Figure 1:**
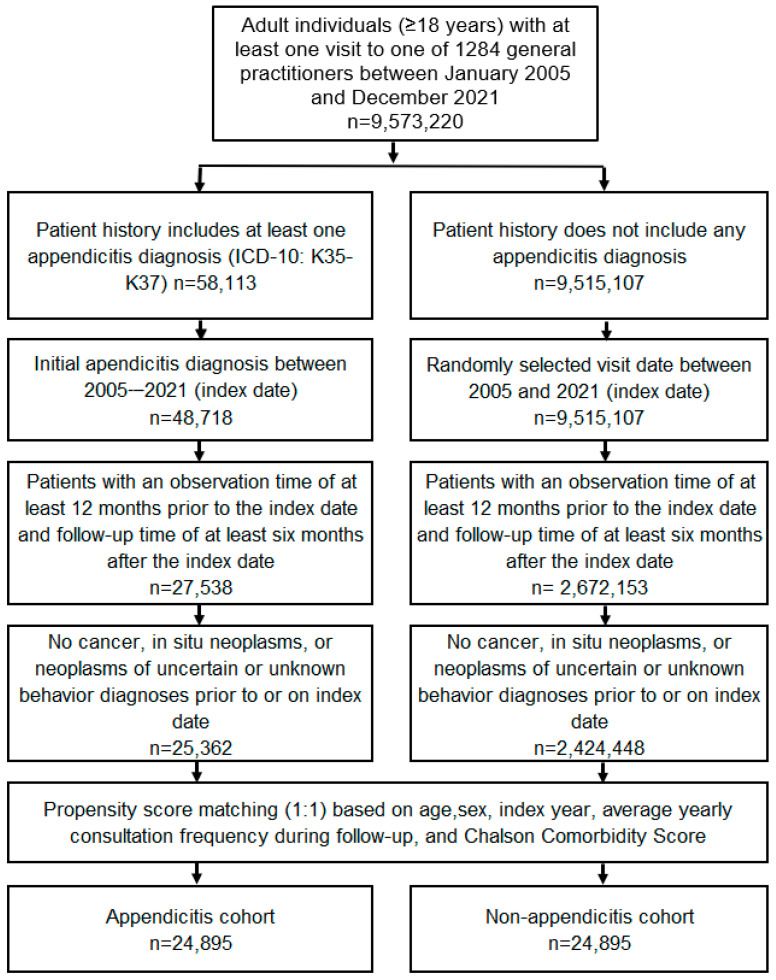
Selection of study patients.

The study included adult patients (≥18 years) with a first diagnosis of appendicitis (ICD-10: K35–K37) in 1284 general practices in Germany between January 2005 and December 2021 (index date; Figure 1). The inclusion criteria required an observation period of at least twelve months prior to the index date and a follow-up period of at least six months after. Patients with diagnoses of cancer (ICD-10: C00–C97), in situ neoplasms (ICD-10: D00–D09), and neoplasms with unclear or unknown diagnosis (ICD-10: D37–D48) before or on the index date were excluded. Individuals without appendicitis matched using propensity score matching (1:1) based on age, sex, index year, average annual consultation frequency during follow-up, and Charlson Comorbidity Score served as the control group. The Charlson index is a weighted index that takes into account the number and severity of comorbidities in administrative database studies and includes a wide range of such conditions (macrovascular disease, lung disease, gastrointestinal, liver and kidney disease, diabetes, AIDS, and others) [26].

The index date for the non-appendicitis cohort was that of a randomly selected visit between January 2005 and December 2021 (Figure 1).

In an initial analysis, the occurrence of cancer (in both groups) was recorded at any time after the index date.

In a second analysis, cancer cases within the first three and six months after the index date were excluded in order to avoid confounding factors and to be able to better verify causal relationships. This makes it easier to interpret the time intervals between the initial diagnosis of appendicitis and the subsequent diagnosis of colon cancer. This makes it possible to better estimate the development times of tumors.

## 3. Results

### 3.1. Basic Characteristics of the Study Sample

This study included 24,895 individuals with appendicitis and 24,895 without appendicitis. A summary of the basic characteristics of the study population is shown in Table 1. The mean age was 41.0 (SD: 17.7) years and 55.6% were female. On average, patients visited their doctor 6.0 times per year during the follow-up period. The median observation period was 5.4 (SD: 3.9) years.

### 3.2. Association between Appendicitis and Subsequent Colon Cancer Diagnosis

**Figure 2:**
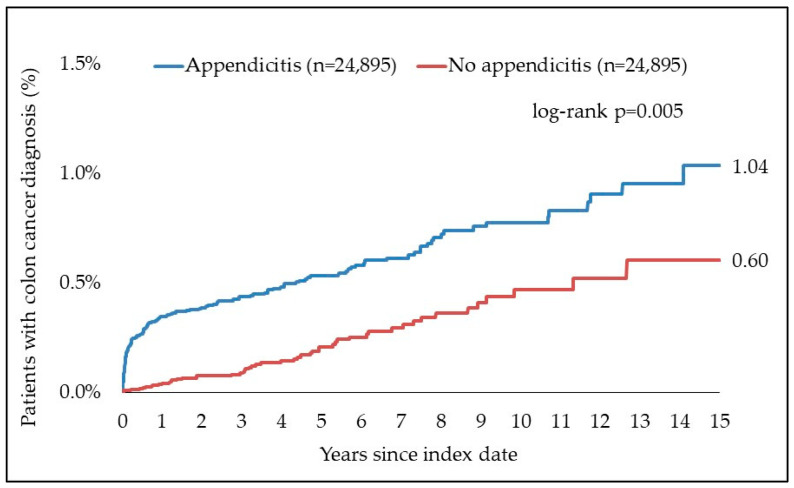
Cumulative incidence of colon cancer in patients with and without appendicitis.

After a follow-up period of up to 15 years, 1.04% of the appendicitis cohort and 0.60% of the non-appendicitis cohort were diagnosed with colon cancer (Figure 2). A significant number of cancer diagnoses (36%) were performed within the first six months of the index date. The largest increase in new diagnoses of colon cancer was observed on days 11, 12 and 25. There were 4 diagnoses per day from the appendicitis cohort.

**Figure 3:**
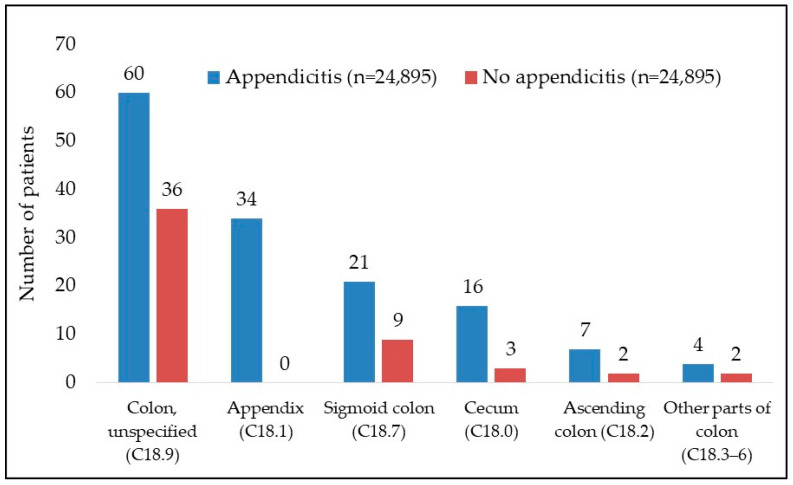
Location of colon cancer in patients with and without appendicitis.

A further breakdown of the observation periods showed that almost half the cancer diagnoses (49%) were performed within 12 months after the index date, while a further 22% were performed more than 5 years after the index date. Table 2 and Table 3 give an overview of the intervals between the index date and diagnosis of colon cancer. Figure 3 shows the location of colon cancer.

The most frequent location was the appendix (34 vs. 0), followed by the sigmoid (21 vs. 9), caecum (16 vs. 3), and ascending colon (7 vs. 2). In most cases, however, the exact location was unknown (Colon unspecified).

The regression analysis showed a significant association between appendicitis and subsequent colon cancer (HR: 2.51; 95% CI: 1.83–3.45). In the age-stratified analysis, the hazard ratio was greatest in the age groups 41–50 (HR: 10.30; 95% CI: 2,42–43.82) and 18–30 years (HR: 8.17; 95% CI: 1.03–64.58). There was also an increased HR for patients > 60 years old (HR: 2.24; 95% CI 1.46–3.45). Furthermore, a stronger effect was observed for men (HR: 3.56; 95% CI: 2.15–5.89) than for women (HR: 1.91; 95% CI: 1.26–2.89) (Table 4).

After excluding carcinoma diagnoses in the initial three months after the index date, there was still a significant increase in the age group 41–50 (HR: 5.34; 95% CI: 1.19–23.86) and in male patients (HR: 2.22; 95% CI 1.30–3.78) compared to the control group. If only carcinoma diagnoses performed after the first 6 months following the index date are considered, the association is only significant for male patients (HR: 2.18; 95% CI 1.26–3.77).

## 4. Discussion

The present study shows a significant association between appendicitis and the subsequent diagnosis of colon cancer. This is in line with previous studies [3,5,6,7], which mostly focused on older patients. We also observed a significant association in the age group >60 years, despite the relative rarity of appendicitis in this age group. Given the elevated incidence of colon cancer within this age range, the 2.24-fold higher HR holds relevance in terms of the absolute number of cancer cases.

However, this study also found a particularly increased HR for the age groups 18–30 and 41–50 years. Interestingly, the association between appendicitis and the diagnosis of colon cancer seemed especially relevant for the male subgroup at all time points investigated.

The correlation between appendicitis and cancer is predominantly noticeable within the initial three months following an appendicitis episode. Nearly half of the cancer diagnoses occurred within the first year after appendicitis, with 36.4% being identified within the initial six months following the index date. Therefore, in a significant proportion of these cases, it can be assumed that appendicitis is the first symptom of still-undiagnosed carcinoma. In such cases, the obstruction of the intestinal lumen by the tumor itself or as part of an inflammatory process in the area of the tumor might have caused appendicitis [16]. This is supported by the finding that, when compared to the group without appendicitis, the rate of malignant neoplasia is particularly high in the parts of the bowel close to the appendix, and especially in the appendix itself (34 vs. 0 cancer diagnoses). While primary malignant tumors of the appendix are generally rare (incidence of 1.2 per 100,000 years) [27], they accounted for almost a quarter of all carcinomas detected in the patients with appendicitis.

However, the increased rate of malignancies in our cohort is not limited to the appendix and the right-sided colon but also affects the sigmoid colon. It has been hypothesized that a malignant process in the distal colon can induce hyperplasia of the lymph follicles in the appendix, potentially leading to the development of appendicitis [20].

According to current guidelines, appendectomy can be dispensed within cases of uncomplicated appendicitis and corresponding imaging suspicion in the age group <65, and conservative treatment can be used primarily [18].

While cancer of the appendix itself will probably be discovered following appendectomy, cancer in more distal parts of the colon might be missed—especially as the symptoms of appendicitis or acute abdomen might dominate, or symptoms caused by the tumor might be misinterpreted as being related to appendicitis or an appendectomy [17,28].

This raises the question of whether patients with appendicitis should be offered regular follow-up care. While in Germany, colorectal cancer screening is covered by statutory health insurance for individuals from the age of 50 with a stool test for both sexes and with a colonoscopy for men aged 50 and over and women aged 55 and over [29]; a follow- up after appendicitis seems especially relevant in young patients whose relative risk of colon cancer is high, risk of endoscopy is low and who are not covered by colon cancer screening.

While we discussed how colonic cancer may lead to appendicitis, others argue that appendicitis might also favor the development of carcinoma due to a disturbance of the microbiome triggered by appendicitis or appendectomy.

Wu and colleagues found a significantly increased incidence of colon carcinoma (HR: 2.13; 95% CI 1.63–2.77) and an earlier manifestation of the condition (i.e., 1.5–3.5 years after appendectomy) in patients after appendicitis than in their control group. In our cohort, however, there is no evidence of an elevated incidence of colon cancer more than 6 months after appendicitis in general, nor in the proposed interval of 1.5–3.5 years after appendicitis. This is in line with the finding that the microbiome composition after appendectomy is not significantly different from the compositions of the microbiome of the inflamed and non-inflamed appendix [30]. However, it is possible that longer follow-up would have been necessary for this observation.

The association between appendicitis and colon cancer has already been investigated in smaller retrospective, mainly monocentric, studies and one prospective Dutch study [14,15,16,17,19,20,31,32]. Nevertheless, our study is of significant value because it is a large cohort representing general practices in Germany. With a few exceptions, previously published results are limited to the Austro-Asian region [14,16,19,21]. Furthermore, patient age was often limited to a fixed age range in other studies [15,16,17,21,33,34,35,36].

In a critical review of our study, the retrospective and non-randomized study design must be mentioned as a limitation. Furthermore, no information is available on treatment for appendicitis, family history of colon cancer nor the lifestyle of the study population with regard to diet, alcohol consumption, smoking, socioeconomic status, and activity profile, even though the Charlson index takes into account the number and severity of comorbidities such as macrovascular disease, lung disease, gastrointestinal, liver and kidney disease, diabetes or AIDS. The potentiation of individual risk factors to an increased inflammatory environment and an increased occurrence and accumulation of mutations that may favor tissue degeneration cannot be assessed. Therefore, the extent to which this could lead to an earlier onset of colorectal cancer cannot be estimated. The possibility that diagnoses were incorrectly assigned during the coding process cannot be ruled out.

To summarize, our study shows a significant association between appendicitis and the subsequent diagnosis of colon cancer. However, further studies are needed to clarify the exact mechanisms and, above all, a possible causal relationship and risk factors.

## 5. Conclusions

On the basis of our data, we assume that, in a relevant number of cases, appendicitis was a symptom of the already-existing colorectal cancer. As described, obstruction by the tumor mass itself, by an inflammatory reaction or by the hyperplasia of the lymphoid follicles are possible causes. The significantly increased tumor rate is not limited to the appendix itself. Based on our results, we recommend offering a colonoscopy or at least a stool test within 12 months after appendicitis, especially to 18–50-year-olds and >60-year-olds in good general health who did not participate in colon cancer screening, particularly to men.

In order to gain a better insight into the causality of tumorigenesis, it is necessary to include multiple risk factors in the analysis. In particular, it is necessary to determine to what extent the presence of several risk factors at the same time may have an effect on the faster development of carcinomas in the context of potentiation.

## Figures and Tables

**Table 1:** Baseline characteristics of the study sample (after 1:1 matching).

Variable	Proportion among Appendicitis Patients (N, %) N = 24,895	Proportion among Non-Appendicitis Patients (N, %) N = 24,895	*p*-Value
Age (Mean, SD)	41.0 (17.7)	41.0 (17.7)	1.000
Age 18–30	9134 (36.7)	9134 (36.7)	1.000
Age 31–40	4332 (17.4)	4332 (17.4)	
Age 41–50	3930 (15.8)	3930 (15.8)	
Age 51–60	3555 (14.3)	3555 (14.3)	
Age > 60	3944 (15.8)	3944 (15.8)	
Female	13,846 (55.6)	13,846 (55.6)	1.000
Male	11,049 (44.4)	11,049 (44.4)	
Number of physician visits per year during the follow-up (Mean, SD)	6.0 (3.8)	6.0 (3.8)	1.000
Charlson Comorbidity Score (CCS)	1.0 (1.4)	1.0 (1.4)	1.000
CCS 0	11,118 (44.7)	11,118 (44.7)	1.000
CCS 1	7733 (31.1)	7733 (31.1)	
CCS 2	3104 (12.5)	3104 (12.5)	
CCS 3	1474 (5.9)	1474 (5.9)	
CCS > 3	1466 (5.9)	1466 (5.9)	
Index year 2005–2008	2595 (10.4)	2595 (10.4)	1.000
Index year 2009–2012	4604 (18.5)	4604 (18.5)	
Index year 2013–2016	6725 (27.0)	6725 (27.0)	
Index year 2017–2021	10,971 (44.1)	10,971 (44.1)	

Proportions of patients given in N, % unless otherwise indicated. SD: standard deviation.

**Table 2:** Time to cancer diagnosis.

Time to Cancer Diagnosis	N	%
≤6 months	71	36.4%
7–12 months	25	12.8%
13–24 months	16	8.2%
25–36 months	11	5.6%
37–60 months	29	14.9%
61–120 months	34	17.4%
>120 months	9	4.6%
Total	195	

**Table 3:** Time to cancer diagnosis excluding the first 6 months.

Time to Cancer Diagnosis	N	%
7–12 months	25	20.2%
13–24 months	16	12.9%
25–36 months	11	8.9%
37–60 months	29	23.4%
61–120 months	34	27.4%
>120 months	9	7.3%
Total	124	

**Table 4:** Association between appendicitis and subsequent colon cancer diagnosis in patients followed in general practices in Germany (univariable Cox regression models).

	Colon Cancer (Diagnoses Starting from Day 1 after Index Date)		Colon Cancer (Excluding Cases Documented within 3 Months after the Index Date)		Colon Cancer (Excluding Cases Documented within 6 Months after the Index Date)	
Subcohort	HR (95% CI)	*p*-Value	HR (95% CI)	*p*-Value	HR (95% CI)	*p*-Value
Total	2.51 (1.83–3.45)	<0.001	1.45 (1.02–2.07)	0.039	1.34 (0.93–1.93)	0.111
Age 18–30	8.17 (1.03–64.58)	0.046	5.21 (0.63–43.32)	0.127	4.23 (0.49–36.30)	0.188
Age 31–40	3.77 (0.80–17.81)	0.093	1.33 (0.22–7.98)	0.759	1.32 (0.21–7.97)	0.759
Age 41–50	10.30 (2.42–43.82)	0.002	5.34 (1.19–23.86)	0.029	4.36 (0.95–19.90)	0.058
Age 51–60	1.61 (0.90–2.88)	0.108	0.85 (0.43–1.68)	0.640	0.84 (0.42–1.70)	0.628
Age > 60	2.24 (1.46–3.45)	<0.001	1.41 (0.87–2.27)	0.164	1.30 (0.80–2.11)	0.288
Female	1.91 (1.26–2.89)	0.002	0.97 (0.59–1.59)	0.912	0.85 (0.51–1.41)	0.527
Male	3.56 (2.15–5.89)	<0.001	2.22 (1.30–3.78)	0.003	2.18 (1.26–3.77)	0.005

## Data Availability

Dataset available on request from the authors. The raw data supporting the conclusions of this article will be made available by the authors on request.
